# Paraneoplastic Endocrine Changes in Gastrointestinal Tumors: A Clinical and Mechanistic Review

**DOI:** 10.3390/ijms27114677

**Published:** 2026-05-22

**Authors:** Dragoș Forțofoiu, Victor-Mihai Sacerdoțianu, Robert-Emmanuel Șerban, Petrică Popa, Ioana-Gabriela Dragne, Ion Rogoveanu, Mihail Virgil Boldeanu, Dragoș-Marian Popescu, Cristin-Constantin Vere

**Affiliations:** 1 Doctoral School, University of Medicine and Pharmacy of Craiova, 200349 Craiova, Romania; dragos.fortofoiu@umfcv.ro (D.F.); gabriela.dragne@umfcv.ro (I.-G.D.); 2 Department of Gastroenterology, University of Medicine and Pharmacy of Craiova, 200349 Craiova, Romania; mihai.sacerdotianu@umfcv.ro (V.-M.S.); ion.rogoveanu@umfcv.ro (I.R.); cristin.vere@umfcv.ro (C.-C.V.); 3 Research Center of Gastroenterology and Hepatology, University of Medicine and Pharmacy of Craiova, 200638 Craiova, Romania; 4 Department of Immunology, University of Medicine and Pharmacy of Craiova, 200349 Craiova, Romania; mihail.boldeanu@umfcv.ro; 5 Department of Extreme Conditions Medicine, University of Medicine and Pharmacy of Craiova, 200349 Craiova, Romania; dragos.popescu@umfcv.ro

**Keywords:** paraneoplastic endocrine syndromes, gastrointestinal malignancies, ectopic hormone secretion, endocrine oncology, Cushing syndrome, SIADH

## Abstract

Paraneoplastic endocrine syndromes (PESs) are hormonal disturbances associated with malignancies that result from tumor-related production of hormone-like substances, immune-mediated mechanisms, or dysregulated signaling pathways. While they are well recognized in lung and neuroendocrine cancers, their relevance in gastrointestinal tumors remains less clearly defined. This narrative review synthesizes current knowledge on paraneoplastic endocrine manifestations in gastrointestinal malignancies, based on a structured search of the literature in major databases, including PubMed, Scopus, and Web of Science. The analysis focuses on clinically relevant syndromes such as hypercalcemia, Cushing-like manifestations, disorders of water balance, hypoglycemia, and acromegaly, with emphasis on underlying mechanisms, associated tumor types, diagnostic approaches, and therapeutic considerations. Available evidence indicates that gastrointestinal tumors can produce a range of biologically active substances, leading to diverse endocrine manifestations that may precede tumor detection and influence disease course. Among these, hypercalcemia and Syndrome of Inappropriate Antidiuretic Hormone Secretion (SIADH) are among the most frequently reported, while other syndromes, such as ectopic Cushing syndrome or tumor-related hypoglycemia, are less common but often associated with more severe clinical outcomes. Recognition of these manifestations has direct clinical implications, as they may support earlier diagnosis, contribute to prognostic assessment, and guide therapeutic management. Improved awareness and a multidisciplinary approach remain essential for optimizing outcomes in patients with gastrointestinal malignancies.

## 1. Introduction

Paraneoplastic endocrine syndromes (PESs) are rare disorders that arise when tumors produce bioactive substances leading to hormonal disturbances in the absence of direct tumor invasion or metastatic involvement of endocrine organs [1,2]. These syndromes pose distinct diagnostic challenges and may precede the detection of an underlying malignancy, thereby serving as early clinical indicators of cancer [1,3].

PESs have been most frequently described in association with lung, thymic, and neuroendocrine malignancies. However, growing evidence indicates that they may also occur in patients with gastrointestinal (GI) cancers, including malignancies of the pancreas, esophagus, stomach, colon, and liver. The underlying mechanisms involve ectopic hormone production or secretion of hormone precursors by tumor cells, driven by altered gene expression. These processes may act through systemic, paracrine, or autocrine pathways, often involving cytokines or hormone-like peptides. Such manifestations may occur in both neuroendocrine and non-neuroendocrine gastrointestinal tumors, although the underlying mechanisms differ [3]. In neuroendocrine tumors, hormone secretion is typically related to the tumor’s intrinsic secretory capacity, whereas in non-neuroendocrine malignancies, it more often reflects aberrant gene expression or dysregulated signaling pathways. It is important to distinguish between neuroendocrine tumors (NETs) and non-neuroendocrine gastrointestinal malignancies in this context. NETs arise from cells with neuroendocrine differentiation and the capacity to secrete hormones, amines, or peptides, making hormone secretion a direct reflection of their intrinsic secretory phenotype. In contrast, paraneoplastic endocrine syndromes in non-neuroendocrine tumors are generally related to ectopic or inappropriate hormone production, representing a dysregulated and non-physiological process. This distinction has important implications for both pathophysiological understanding and clinical management. Clinically, PESs can present severe metabolic disturbances, such as hypercalcemia or hyponatremia, which are frequently associated with an unfavorable prognosis.

Despite the increasing recognition of paraneoplastic endocrine syndromes (PESs), their occurrence in gastrointestinal malignancies remains incompletely characterized [1]. Most available studies focus on individual syndromes or specific tumor types, whereas broader overviews of gastrointestinal cancers are still limited. In addition, recent advances regarding molecular mechanisms and diagnostic approaches have not always been incorporated into a clinically oriented perspective. As a result, these syndromes continue to represent an important diagnostic and therapeutic challenge in gastrointestinal oncology.

This review brings together current knowledge on paraneoplastic endocrine disturbances in GI tumors using a syndrome-based perspective. It outlines the main pathogenic mechanisms, clinical manifestations, diagnostic considerations, and therapeutic options for the major PESs encountered in this context. Particular attention is given to the mechanisms underlying ectopic hormone secretion and to the spectrum of gastrointestinal tumors associated with each syndrome. To support clinical applicability, summary tables are included to correlate tumor types, secreted factors, and associated clinical features. By organizing the available data in this manner, the review aims to facilitate earlier recognition, improve diagnostic accuracy, and support multidisciplinary management in patients with gastrointestinal malignancies.

In this context, the present review aims to provide a comprehensive and clinically oriented synthesis of paraneoplastic endocrine syndromes associated with gastrointestinal malignancies, integrating current evidence on pathophysiological mechanisms, clinical manifestations, diagnostic strategies, and therapeutic approaches.

## 2. Materials and Methods

### 2.1. Review Design

The present work constitutes a narrative review of the scientific literature, developed with the objective of systematically synthesizing current evidence on paraneoplastic endocrine syndromes (PESs) associated with gastrointestinal (GI) malignancies. The review was structured around a syndrome-oriented framework, addressing each major PES through pathophysiological mechanisms, clinical presentation, diagnostic approach, and therapeutic management. The review follows the general methodological principles applicable to narrative reviews in clinical medicine, with a focus on mechanistic and clinical evidence integration rather than statistical pooling.

### 2.2. Data Sources and Search Strategy

A comprehensive search of peer-reviewed biomedical literature was conducted across the following electronic databases: PubMed/MEDLINE, Scopus, Web of Science (Science Citation Index Expanded—SCIE), and EMBASE. These platforms were selected for their extensive and complementary coverage of journals in endocrinology, oncology, gastroenterology, and related disciplines. Additional sources consulted included reference lists of retrieved articles, clinical practice guidelines (ESC, ENDO, ERS, KDIGO), endocrinological society consensus documents, and PubMed Central (PMC) for open-access full-text retrieval. The online supplementary database AACE Clinical Case Reports and Endocrinology, Diabetes & Metabolism Case Reports were also screened for relevant clinical cases.

The search spanned 39 years, from 1984 to 2023, covering publications. The earliest reference dates from 1984 (Vassilopoulou-Sellin et al., Cancer), while the most recent source was published in 2023 (Mac Eochagain et al., Annals of Esophagus). The search terms were structured using Boolean operators (AND, OR) around the following core Medical Subject Headings (MeSH) and free-text keywords:

- Primary terms: “paraneoplastic syndrome”, “ectopic hormone secretion”, “ectopic ACTH”, “ectopic hormone production”, “paraneoplastic endocrine”
- Tumor-related terms: “gastrointestinal tumors”, “colorectal carcinoma”, “gastric cancer”, “pancreatic neuroendocrine tumor”, “hepatocellular carcinoma”, “esophageal carcinoma”, “gastrointestinal stromal tumor” (GIST)
- Syndrome-specific terms: “hypercalcemia of malignancy”, “PTHrP”, “Cushing syndrome”, “SIADH”, “hyponatremia”, “non-islet cell tumor hypoglycemia”, “NICTH”, “IGF-2”, “acromegaly”, “GHRH”, “gynecomastia”, “paraneoplastic”, “ectopic TSH”, “ectopic calcitonin”, “ectopic prolactin”
- Therapeutic/diagnostic terms: “somatostatin analogs”, “bisphosphonates”, “tolvaptan”, “metyrapone”, “ketoconazole”, “68Ga-DOTATATE PET”, “octreotide scintigraphy”, “endocrine oncology”

No language restriction was applied during the initial search phase; however, all articles included in the final analysis were published in English, except for reference [4] (Arai et al., published in Japanese in Gan To Kagaku Ryoho, 2010), which was included based on its abstract and clinical data.

### 2.3. Inclusion and Exclusion Criteria

#### 2.3.1. Inclusion Criteria

- Studies reporting paraneoplastic endocrine manifestations in patients with histologically confirmed gastrointestinal malignancies, including tumors of the esophagus, stomach, small intestine, pancreas, colon, rectum, and liver.
- Publications addressing at least one of the following syndromes: hypercalcemia of malignancy (PTHrP- or calcitriol-mediated), ectopic ACTH-dependent Cushing syndrome, syndrome of inappropriate antidiuretic hormone secretion (SIADH), non-islet cell tumor hypoglycemia (NICTH/big IGF-2), ectopic GHRH-mediated acromegaly, paraneoplastic gynecomastia (hCG/estrogen-mediated), ectopic TSH secretion, ectopic prolactin, or ectopic calcitonin production.
- Articles providing clinically or mechanistically relevant data on pathogenesis, diagnostic evaluation, or therapeutic management.
- Study types accepted: original research articles, systematic reviews, narrative reviews, clinical practice guidelines, consensus documents, cohort studies, case series, and case reports.
- Publications from 1984 to 2023 (inclusive).

#### 2.3.2. Exclusion Criteria

- Studies focusing exclusively on paraneoplastic syndromes arising from non-gastrointestinal primary tumors (small-cell lung carcinoma, thymoma, pituitary adenoma, etc.) without direct relevance to GI oncology.
- Articles reporting endocrine abnormalities attributable to direct tumor invasion of endocrine organs, skeletal metastases, or primary endocrine disease, rather than ectopic hormone secretion.
- Publications without sufficient clinical or mechanistic data to contribute meaningfully to the review objectives.
- Letters, editorials, and conference abstracts without original data.

### 2.4. Article Selection and Data Extraction

Titles and abstracts were independently screened by the review authors for relevance. Full-text articles were subsequently retrieved and assessed against the predefined inclusion and exclusion criteria. For each included publication, the following information was systematically extracted and organized by syndrome: the implicated gastrointestinal tumor type, any identified ectopic hormone or mediator, the described pathophysiological mechanism, the reported clinical and biochemical features, the diagnostic workup performed, and the therapeutic interventions applied. Case reports were incorporated to illustrate characteristic clinical presentations and atypical management scenarios. Extracted data were synthesized narratively and tabulated in two summary tablescorrelating GI tumor types, ectopic hormones, and resultant syndromes.

### 2.5. Quality Assessment

Given the predominantly observational and descriptive nature of the available evidence—reflecting the clinical rarity of paraneoplastic endocrine syndromes in gastrointestinal oncology—formal risk-of-bias assessment tools such as the Newcastle-Ottawa Scale, the Cochrane Risk of Bias tool, or the GRADE framework were not applied in their standard format. The available literature consists largely of case reports, small case series, and narrative reviews, with only a limited number of prospective cohort studies and randomized controlled trials. The level and consistency of evidence supporting each clinical and mechanistic statement were critically evaluated during data synthesis, with particular attention to concordance across multiple independent publications and to the mechanistic plausibility of the reported findings.

No statistical meta-analysis was performed. A formal PRISMA flow diagram was not generated, as this review does not constitute a systematic review in the strict methodological sense. Nevertheless, the methodological approach ensures transparency in source identification and data selection, in accordance with the standards of evidence-based narrative reviews in clinical medicine.

## 3. Paraneoplastic Endocrine Syndromes Associated with Gastrointestinal Malignancies

### 3.1. Hypercalcemia of Malignancy

#### 3.1.1. Epidemiology and Real Incidence

Hypercalcemia of malignancy (HCM) has been classically cited as affecting up to 30% of cancer patients at some point during their disease progression [5]. However, this estimate, derived from small cohorts with incomplete diagnostic evaluation, is no longer supported by contemporary population-level data. A landmark retrospective cohort study by Sheehan et al., encompassing 167,551 adult oncological patients followed for 20 years, identified hypercalcemia in 6.9% of patients and confirmed HCM in only 0.67–2.0% of the total cancer population [6]. This discrepancy has important clinical implications: it signals that HCM should not be assumed in every hypercalcemic cancer patient, and that the true mechanistic distribution differs substantially from historical teaching. Despite its lower-than-expected incidence, HCM remains a metabolic emergency. It is characterized by its potential to trigger acute metabolic crises and its association with advanced malignancy and poor clinical outcomes [5,7,8].

#### 3.1.2. Pathophysiological Mechanisms Beyond PTHrP Monism

The fundamental mechanism in most gastrointestinal malignancies is ectopic production of parathyroid hormone-related peptide (PTHrP), which reproduces the biological effects of parathyroid hormone (PTH) by promoting osteoclast-mediated bone resorption and enhancing renal tubular calcium reabsorption. Additional contributing processes include excessive production of 1,25-dihydroxyvitamin D and pro-inflammatory cytokines, such as IL-1 and TNF-α [5,7,8].

The canonical model assigns PTHrP-mediated humoral hypercalcemia as the dominant mechanism in approximately 80% of solid tumor HCM cases. This construct has been progressively challenged by studies employing comprehensive simultaneous biochemical evaluation. In the Sheehan et al. cohort, among 70 patients with a fully triangulated workup (PTH, PTHrP, 1,25 OH vitamin D, skeletal imaging), the mechanistic distribution was: PTHrP 27%, osteolytic metastases 50%, calcitriol elevation 39%, with combined mechanisms present in approximately 20% of cases [6]. These proportions overturn the classical hierarchy and identify calcitriol co-elevation as a far more common contributor than previously recognized.

Four mechanistic pathways are currently established:

1. PTHrP mediated humoral hypercalcemia (HHM): PTHrP mimics PTH at the PTH/PTHrP receptor (PTH1R), stimulating osteoclastogenesis via the RANK RANKL axis, promoting renal tubular calcium reabsorption, and suppressing intact PTH through negative feedback. The PTHLH gene encoding PTHrP is activated in malignant cells through multiple oncogenic pathways, including Ras/MAPK, TGF-β, and Wnt/β-catenin signaling. In squamous cell carcinomas, this activation is particularly robust due to baseline low-level PTHLH expression in stratified squamous epithelium [5,7,8].
2. Calcitriol mediated (ectopic 1α hydroxylase) hypercalcemia: Malignant cells may express CYP27B1 (1α hydroxylase), converting 25 OH vitamin D to the active 1,25 OH vitamin D, which enhances intestinal calcium absorption. This pathway has been confirmed in gastrointestinal squamous and adenocarcinomas [6].
3. Osteolytic metastasis-driven hypercalcemia: Local osteoclast activation by tumor-secreted cytokines (IL-1β, IL-6, TNF-α, RANKL) at skeletal metastatic sites. Although typically associated with breast cancer and multiple myeloma, osteolytic HCM occurs in advanced GI malignancies with bone involvement [5,8].
4. Co-mediated hypercalcemia: The co-occurrence of PTHrP elevation and calcitriol overproduction in the same patient creates therapeutic complexity, as bisphosphonates are insufficient in isolation. Song et al. (2021) documented the first case of co-mediated HCM in esophageal squamous cell carcinoma and proposed a revised diagnostic algorithm that mandates simultaneous measurement of PTHrP, 25-OH vitamin D, and 1,25-OH vitamin D in all cases of PTH-independent hypercalcemia [9,10,11].

#### 3.1.3. Tumor Type Specificity in Gastrointestinal Malignancies

Hypercalcemia has been documented in various gastrointestinal malignancies, including esophageal squamous cell carcinoma, gastric adenocarcinoma, colorectal carcinoma, and hepatocellular carcinoma, and is associated with poor prognosis [5,9,10,11,12]. Although it is not a specific feature of these malignancies, its occurrence should raise concern of a paraneoplastic condition, especially in the absence of bone metastases. Critically, extrapolating data from lung or breast cancer cohorts to GI oncology is methodologically inappropriate. Based on available primary evidence, the following tumor-specific patterns can be delineated:

Esophageal squamous cell carcinoma (ESCC): PTHrP is the dominant mediator, consistent with the squamous histotype’s intrinsic PTHLH expression capacity. Song et al. (2021) demonstrated that co-mediation of HCM (PTHrP + calcitriol) is possible in ESCC and poses specific therapeutic challenges: bisphosphonates only partially correct calcium, while glucocorticoids are additionally required to suppress ectopic 1α hydroxylase activity [11]. In the Donovan et al. retrospective series of 138 PTHrP-mediated HCM patients, solid organ malignancies, including squamous carcinomas, carried a median survival of only 52 days (IQR 21–132 days) [13].

Gastric adenocarcinoma: PTHrP production has been confirmed immunohistochemically in gastric carcinoma cells. Unlike ESCC, gastric cancer may produce PTHrP even in the absence of bone metastases, and hypercalcemia can present as an early paraneoplastic signal that precedes or accompanies the oncological diagnosis [5,9].

Colorectal carcinoma (CRC): HCM is rare in CRC but clinically severe when present. Galindo et al. (2016) conducted a systematic analysis of 29 published cases of PTHrP-mediated HCM in colorectal cancer, combined with an original case report [14]. Key findings: patients were predominantly middle-aged men (mean age 56.7 ± 13.4 years), with 85% having hepatic metastases and 62% presenting with severe hypercalcemia (Ca > 14 mg/dL). Mortality was 79%, with a median time to death of 54.5 days (CI: 21–168). Response to bisphosphonates was poor; chemotherapy aimed at tumor control yielded the most meaningful reduction in calcium. Combined elevation of PTHrP and calcitriol was documented in individual cases, consistent with the co-mediated model [14].

Hepatocellular carcinoma (HCC): HCM in HCC is mechanistically heterogeneous. Beyond PTHrP secretion, extensive parenchymal replacement impairs hepatic calcium homeostasis, and there is potential coactivation of ectopic 1α hydroxylase. The simultaneous disruption of multiple calcium-regulating pathways explains the frequent occurrence of bisphosphonate resistance in HCC-associated HCM [5,12].

Pancreatic neuroendocrine tumors (pNETs): PTHrP-secreting pNETs, although rare, represent a diagnostically challenging entity. In these tumors, PTHrP secretion may be controlled by somatostatin analog therapy, offering a specific therapeutic option not available in non-neuroendocrine HCM [5].

#### 3.1.4. Clinical Manifestations

Clinical manifestations may include nausea, vomiting, constipation, polyuria, dehydration, and disorientation, while severe cases can progress to cardiac arrhythmias and coma. These manifestations are often non-specific and may be easily missed in patients with advanced cancer [5,8,13]. HCM severity correlates with both the absolute calcium level and the rapidity of onset, making early biochemical detection.

#### 3.1.5. Diagnostic Approach

The diagnostic approach involves confirming elevated blood calcium levels, with suppressed intact PTH and elevated PTHrP levels. Measuring 1,25-dihydroxyvitamin D levels can aid in distinguishing between PTHrP and vitamin D-mediated hypercalcemia. The absence of skeletal lesions on imaging may point toward a paraneoplastic etiology [7,13,15]. Critically, a major limitation of current clinical practice documented by the Sheehan et al. cohort is that only 25.4% of hypercalcemic cancer patients had PTH measured, and the full triangulated workup was completed in a minority [6]. This diagnostic incompleteness results in misclassification of mechanism and suboptimal treatment selection.

The recommended stepwise algorithm includes: (1) corrected serum calcium + intact PTH to confirm PTH independence; (2) PTHrP measurement; (3) simultaneous 1,25 OH vitamin D (calcitriol) mandatory in all cases, not only when PTHrP is normal, as co elevation changes the treatment strategy; (4) 25 OH vitamin D baseline; (5) skeletal imaging to identify osteolytic contribution [6,10].

#### 3.1.6. Therapeutic Approach

The initial therapeutic approach focuses on stabilizing the patient with intravenous fluid administration and bisphosphonate therapy, which suppresses osteoclast-mediated bone resorption [15]. Calcitonin can be administered to rapidly decrease calcium levels, but only temporarily [. Denosumab, a RANKL inhibitor, has been effective in refractory cases [16,17,18]. In co-mediated HCM (PTHrP + calcitriol), bisphosphonates alone are insufficient, and the addition of glucocorticoids to suppress ectopic CYP27B1 activity is required, a treatment nuance systematically missed when calcitriol measurement is omitted from the diagnostic workup [6,10]. Definitive management depends on the type and stage of the tumor and involves managing the primary tumor with surgical intervention, chemotherapy, or radiation therapy [5,17,18]. A critical principle supported by both the Ralston et al. and Galindo et al. primary datasets is that antihypercalcemic therapy alone does not confer a survival benefit: calcium correction is a metabolic bridge, not a definitive intervention [12,14].

#### 3.1.7. Critical Analysis Evidence Appraisal

Well supported by primary evidence: PTHrP is the most common single mediator in solid GI tumors, but calcitriol co-elevation is more prevalent than classical teaching suggests [6,14]. Zoledronic acid is superior to pamidronate in HCM (Level I RCT) [15]. Tumor-directed therapy is the only intervention associated with meaningful survival improvement [12]. HCM in colorectal cancer is almost exclusively associated with advanced hepatic metastatic disease and carries a median survival of approximately 54.5 days [14].

Areas of uncertainty and contradictory data: The true incidence of HCM across GI tumor types is unknown because no prospective GI-specific registry exists. Galindo et al. found no statistically significant correlation between PTHrP level and mortality in CRC (*p* = 1.0), which challenges the notion of PTHrP as a prognostic biomarker in this context [14]. The optimal sequencing of bisphosphonates versus denosumab as first-line therapy in GI malignancies has not been established by RCT data. The impact of novel oncological therapies (immune checkpoint inhibitors, targeted agents) on HCM incidence and biology in GI malignancies remains entirely unexplored.

Gaps in the literature: No prospective cohort has specifically characterized HCM in the GI oncology setting using simultaneous comprehensive biochemical evaluation. The pharmacological management of co-mediated HCM lacks RCT evidence, and current recommendations are extrapolated from case series. Whether serial PTHrP monitoring after treatment is a valid biomarker for detecting tumor recurrence in GI cancers has not been prospectively validated.

#### 3.1.8. Case Report

Yamada et al. (2021) reported on a patient with advanced-stage gastric cancer with severe hypercalcemia caused by ectopic PTHrP release, as shown by high serum PTHrP and reduced PTH levels. Management with hydration, bisphosphonates, and tumor-directed therapy corrected the calcium imbalance.

This case illustrates several diagnostically and therapeutically critical principles. First, the biochemical pattern elevated PTHrP, suppressed PTH, and absent skeletal metastases constitutes the defining signature of humoral HCM and should be systematically sought in any gastric cancer patient with unexplained hypercalcemia, regardless of disease stage. Clinicians frequently attribute hypercalcemia in oncological patients to bone metastases by default, thereby missing the paraneoplastic diagnosis and delaying appropriate treatment. Second, the response to bisphosphonates in this case confirms their role as first-line agents in isolated PTHrP-driven HCM; however, calcium normalization without tumor response does not extend survival beyond a median of 30 days, as demonstrated by the Ralston et al. primary cohort [12]. Third, normalization of PTHrP following tumor-directed treatment can serve as a surrogate biomarker of therapeutic response, while a renewed rise in serum calcium or PTHrP in a previously normocalcemic patient should prompt immediate oncological restaging, as endocrine parameters may herald tumor progression before radiological changes become apparent [6,12].

### 3.2. Cushing’s Syndrome (Ectopic ACTH Secretion)

#### 3.2.1. Epidemiology and Prevalence in GI/GEP Tumors

Paraneoplastic Cushing syndrome (PCS) resulting from ectopic ACTH secretion (EAS) accounts for 5–20% of all Cushing syndrome cases and approximately 10–20% of all ACTH-dependent Cushing syndrome cases [19,20]. Its incidence in the general population is estimated at 1–3 new cases per million people per year [21]. Within the specific context of gastroenteropancreatic (GEP) and thoracic neuroendocrine tumors, primary cohort data provide more precise figures. Kamp K, Alwani RA, Korpershoek E et al. prospectively evaluated 918 patients with thoracic or GEP NETs at a tertiary referral center over a 19-year period (1993–2012) and identified EAS in 29 patients, yielding a prevalence of 3.2% [21]. Pancreatic NETs and thoracic carcinoids were the principal GI-related sources. EAS patients exhibited significantly shorter 5-year survival compared to non-EAS NET patients, establishing EAS as an independent prognostic determinant [21].

In a large Italian multicenter cohort, Davi et al. retrospectively analyzed 110 patients with NETs and EAS collected from 17 centers [22]. The main GI-related sources of ectopic ACTH were pancreatic NETs (pNETs), accounting for 15.5% of cases, while bronchial carcinoids dominated at 40.9%, and occult tumors represented 22.7%. Among pNET patients, curative surgical resection was achievable in only 11%, a stark contrast to the 70.2% resectability rate in bronchial carcinoids, reflecting the typically advanced and metastatic stage at EAS presentation in GI tumors [22]. Additionally, Paleń-Tytko JE, Przybylik-Mazurek EM et al., reporting a 20-year single-center experience with 24 EAS patients, demonstrated that GEP NET patients had significantly higher midnight cortisol levels than non-GEP NET patients, and that hypercortisolemia severity correlated with ACTH levels and the degree of hypokalemia, a finding with direct clinical monitoring implications [23].

#### 3.2.2. Pathophysiological Mechanisms

PCS is caused by ectopic production of adrenocorticotropic hormone (ACTH) or, less commonly, of corticotropin-releasing hormone (CRH) by non-pituitary tumor cells. The molecular basis involves aberrant repression of the pro-opiomelanocortin (POMC) gene in tumor cells through epigenetic mechanisms, including hypomethylation of the POMC promoter and activation of transcription factors such as NeuroD1 and Tpit, which are normally restricted to pituitary corticotroph cells [19,20]. Additionally, altered post-translational processing of POMC in ectopic tumors results in elevated secretion of ACTH precursors (pro ACTH, big ACTH) alongside bioactive ACTH, which can be measured to support the ectopic diagnosis.

The sustained hypercortisolemia that follows impairs the hypothalamic–pituitary–adrenal (HPA) axis by downregulating CRH and pituitary ACTH synthesis, while also driving bilateral adrenocortical hyperplasia. This ACTH-independent bilateral adrenal enlargement is a key imaging finding in EAS. In a minority of cases, ectopic CRH production stimulates pituitary ACTH release, creating a biochemical profile that closely resembles Cushing’s disease and significantly complicating the differential diagnosis [19,20].

#### 3.2.3. Tumor Type Specificity in GI Malignancies

PCS is rarely associated with gastrointestinal cancers; it has been reported in NETs of the pancreas and small intestine, and also in non-neuroendocrine GI tumors such as colorectal carcinomas and metastatic hepatocellular carcinoma in atypical cases [19,20,24,25]. A critical distinction, underappreciated in the clinical literature, concerns the mechanistic and prognostic differences between neuroendocrine and non-neuroendocrine GI sources of EAS:

Pancreatic NETs (pNETs): The most clinically significant GI source. pNETs producing ectopic ACTH tend to present at advanced, metastatic stages, with liver involvement being common. The Davi et al. multicenter cohort demonstrated that among pNET EAS patients, curative surgery was possible in only 11% of cases, and overall survival was significantly shorter than in bronchial carcinoid-associated EAS [22]. A particularly important finding from Lase et al. (2020), who analyzed 886 NEN patients and identified 51 with ECS, is that in 12 patients the NEN diagnosis preceded ECS development by a median of 43.5 months (range 9–96 months), with 10 of these 12 showing radiological tumor progression at ECS onset indicating that EAS emergence in a previously non secreting pNET signals disease progression rather than a new independent event [26].

Small bowel NETs (ileal carcinoids): Late onset EAS is characteristic. The case described by Tsagarakis et al. (2020) illustrates that ectopic ACTH production can appear over a year after surgical resection of a low-grade small bowel NET with no increase in tumor burden [25], supporting the concept of metachronous or cyclic EAS, a biological phenomenon linked to epigenetic changes in tumor cells rather than simple tumor progression.

Non-neuroendocrine GI tumors (colorectal carcinoma, HCC): EAS in these contexts is exceedingly rare and typically associated with dedifferentiation or neuroendocrine transdifferentiation of the primary tumor. Clinical presentation is often atypical, with weight loss and cachexia predominating over classic Cushingoid features, as the rapid pace of disease does not allow the phenotypic manifestation of hypercortisolemia [23,24].

#### 3.2.4. Clinical Manifestations

Clinical manifestations of PCS may include central obesity, “moon face” appearance, hypertension, hyperglycemia, muscular weakness, and psychiatric disturbances [20,27]. However, primary cohort data consistently show that typical Cushingoid appearance is uncommon in EAS associated with aggressive or rapidly progressing tumors: in the Paleń-Tytko JE, Przybylik-Mazurek EM et al. series, classical Cushingoid features were present in only a minority of patients, while weight loss was more frequent than weight gain [23]. This paradoxical hypercortisolemia without the phenotypic Cushing habitus is attributable to the dominance of cortisol’s catabolic effects in the context of an advanced malignancy and constitutes the primary reason EAS is underdiagnosed in this population. The biochemical triad of severe hypokalemia, hypertension, and proximal myopathy without overt Cushingoid appearance is a key clinical alert for EAS in GI malignancy patients [19,24].

#### 3.2.5. Diagnostic Approach

ACTH-dependent hypercortisolism is confirmed by demonstrating elevated morning cortisol and ACTH levels, a lack of suppression on high-dose dexamethasone testing (8 mg), and imaging evaluation to rule out a pituitary source [19,24,27]. When the distinction between EAS and Cushing’s disease is biochemically ambiguous, which occurs in approximately 10–15% of cases, inferior petrosal sinus sampling (IPSS) after CRH stimulation is the gold standard, showing a central to peripheral ACTH gradient <2 (basal) or <3 (post CRH) in EAS [20,28]. Chromogranin A and urine 5-HIAA levels may support a NET diagnosis [19,24,27]. For tumor localization, ^68^Ga DOTATATE PET/CT has emerged as the most sensitive modality for somatostatin receptor-expressing tumors [29,30], whereas ^18^F FDG PET/CT is preferred for high-grade or dedifferentiated tumors with low somatostatin receptor expression.

#### 3.2.6. Therapeutic Approach and Evidence Level

The treatment of PCS aims to control cortisol excess and address the underlying tumor. Agents that inhibit adrenal steroidogenesis, such as ketoconazole or metyrapone, are commonly used as first-line medical therapies [30,31]. Etomidate may be administered in hospitalized patients with acute hypercortisolemic crises [31]. For refractory cases, mifepristone, a glucocorticoid receptor antagonist, may be considered [32]. In patients who do not respond to medical therapy, bilateral adrenalectomy represents a definitive but noncurative option for cortisol control [23]. Tumor control through surgical resection, somatostatin analogs, or targeted therapy may reduce ectopic ACTH production [19,24,30]. Primary cohort data from Davi et al. confirm that, when achievable, curative surgery is the only intervention consistently associated with complete biochemical remission and improved survival in this setting [22].

#### 3.2.7. Comparative Overview of Ectopic ACTH Syndrome by GI Tumor Type

Ectopic ACTH syndrome represents a rare but clinically significant paraneoplastic manifestation of gastrointestinal malignancies, characterized by severe hypercortisolism, metabolic disturbances, and frequently atypical clinical presentations. Although neuroendocrine tumors remain the most common gastrointestinal neoplasms associated with ectopic ACTH secretion, increasing evidence suggests that poorly differentiated colorectal carcinoma, hepatocellular carcinoma, and other aggressive gastrointestinal malignancies may also acquire aberrant proopiomelanocortin (POMC) expression and ectopic ACTH production. Importantly, the underlying pathogenic mechanisms, hormonal profiles, imaging characteristics, treatment responsiveness, and clinical outcomes vary considerably according to tumor origin, degree of neuroendocrine differentiation, and disease stage. To provide a comparative overview of the principal clinical, biochemical, radiologic, and therapeutic characteristics associated with ectopic ACTH syndrome in gastrointestinal malignancies, Table 1 summarizes the major features reported across different gastrointestinal tumor types.

#### 3.2.8. Evidence Appraisal

Well supported by primary evidence: EAS prevalence in GEP NETs is approximately 3.2% [21]. Pancreatic NETs are the dominant GI source of EAS, but have low surgical curability (11%) [22]. Hypercortisolemia severity, quantified by midnight cortisol, correlates with clinical severity and hypokalemia [23]. Tumor resection is the only intervention conferring complete biochemical remission [22].

Areas of uncertainty: Whether EAS emergence in a known pNET always signals progression (as suggested by Lase et al.) or can occur without radiological changes (as in the Tsagarakis case [25]) remains debated, with both patterns documented in primary literature. The optimal cortisol-lowering agent (ketoconazole vs. metyrapone vs. combination) in GI EAS has not been evaluated in a controlled trial. The role of timing for bilateral adrenalectomy in unresectable cases lacks prospective validation.

Key gaps: No prospective GI-specific EAS registry exists. The impact of modern oncological therapies (everolimus, sunitinib, PRRT) on EAS in pNETs has been documented anecdotally but not systematically. Data on EAS in non-neuroendocrine GI tumors are limited to isolated case reports.

#### 3.2.9. Case Report

Mineur et al. (2021) reported a 58-year-old patient with a pancreatic NET presenting with paraneoplastic Cushing syndrome associated with severe hypokalemia and hypertension. The diagnosis was established by elevated ACTH and cortisol levels and a suppressed dexamethasone response. Management with metyrapone, followed by treatment using somatostatin analogs, improved metabolic parameters and symptoms [17].

This case illustrates several principles with direct clinical implications. First, the biochemical presentation of severe hypokalemia, hypertension, elevated ACTH, and cortisol with dexamethasone non-suppression is the archetypal signature of EAS in the context of a GI NET, and should immediately orient the diagnostic workup toward imaging for tumor localization, bypassing unnecessary dynamic testing for Cushing’s disease. Second, the case demonstrates the dual therapeutic strategy that is required in GI EAS: cortisol-lowering therapy (metyrapone) as a bridge, combined with tumor-directed treatment (SSA) to reduce ACTH production at the source. This two-pronged approach is supported by the multicenter cohort data of Davi et al., which confirm that biochemical control without tumor remission does not confer a survival benefit [22]. Third, the absence of classic Cushingoid habitus in this patient despite documented hypercortisolemia illustrates a pattern consistently observed in the primary cohort data [23]: in GI malignancy-associated EAS, the tumor’s catabolic burden overrides the anabolic effects of hypercortisolemia, making hypokalemia and muscle weakness the dominant clinical signals. Clinicians must internalize this atypical phenotype to avoid diagnostic delay. Fourth, from a monitoring perspective, metyrapone dose should be titrated to serum cortisol, not to clinical appearance, since phenotypic improvement lags behind biochemical correction, and over-treatment risks adrenal insufficiency, which carries significant morbidity in this immunocompromised population.

### 3.3. Syndrome of Inappropriate Antidiuretic Hormone Secretion (SIADH)

#### 3.3.1. Epidemiology

SIADH is a paraneoplastic syndrome characterized by inappropriate secretion of antidiuretic hormone (ADH/vasopressin), resulting in water retention, euvolemic hyponatremia, and reduced plasma osmolality [33,34]. The primary clinical manifestation of SIADH, the most common electrolyte disorder in hospitalized cancer patients, occurs in 18–47% of this population [35]. Its prognostic significance has been established by primary cohort data with compelling quantitative evidence.

Doshi et al. (2012) conducted a retrospective analysis of prospectively collected data on cancer patients admitted to MD Anderson Cancer Center, demonstrating that hyponatremia was associated with significantly longer hospital stay and higher in-hospital mortality compared to normonatremic patients [35]. The prognostic impact was further quantified in the prospective HYPNOSIS study by Fucà et al. (2019), which enrolled 279 patients with metastatic solid tumors (median follow up 27 months): median survival was 2 months in hyponatremic patients versus 13.2 months in normonatremic patients (HR = 2.65; *p* < 0.001), making hyponatremia one of the most powerful independent prognostic factors in metastatic oncology [36]. Furthermore, the first multicenter Italian study of SIADH in cancer patients by Berardi et al. (2019), enrolling 90 patients from 28 institutions, demonstrated that failure to normalize sodium during hospitalization significantly reduced overall survival, and that tolvaptan-treated patients had shorter hospitalization duration [37].

Within gastrointestinal malignancies, SIADH is most often associated with esophageal squamous cell carcinoma, gastric adenocarcinoma, and pancreatic adenocarcinoma, and is particularly linked to advanced-stage tumors [5,8,9,33,38].

#### 3.3.2. Pathophysiological Mechanisms

Ectopic production of vasopressin (arginine vasopressin, AVP) by tumor cells is the primary mechanism of paraneoplastic SIADH in GI malignancies. AVP binds to V2 receptors in the renal collecting duct, activating adenylyl cyclase, increasing intracellular cAMP, and promoting insertion of aquaporin 2 (AQP2) water channels into the apical membrane. This results in free water retention, plasma dilution, and euvolemic hyponatremia despite suppressed plasma osmolality [38,39].

A critical mechanistic distinction must be made between the different sources of SIADH in GI oncology, which has direct diagnostic and therapeutic implications:

Esophageal squamous cell carcinoma (ESCC): Represents the most frequently documented GI tumor in paraneoplastic SIADH. The squamous epithelium of the esophagus does not constitutively express the AVP gene; however, epigenetic derepression under hypoxic and oncogenic conditions (particularly TP53 mutation and hypermethylation of tumor suppressor loci) can activate ectopic AVP synthesis. SIADH in ESCC is typically a marker of advanced locoregional or metastatic disease [8,9].

Gastric adenocarcinoma: May produce AVP ectopically or may induce SIADH through a cytokine-mediated mechanism (IL-6-stimulated hypothalamic AVP release), creating a biochemically indistinguishable but pathogenetically distinct syndrome [5,8]. Hyponatremia may appear early in the disease course and has been proposed as a potential early diagnostic signal. The case documented by Shimizu et al. (2017) illustrates the clinical severity possible in this context, Na^+^ = 112 mmol/L with neurological compromise [40].

Pancreatic adenocarcinoma: Rare but documented. The case reported by Nagashima et al. (1996) [38] described ectopic ADH production by a pancreatic adenocarcinoma, a non-neuroendocrine tumor, demonstrating that even histologically non-secretory tumors may develop paraneoplastic endocrine activity through aberrant gene expression.

Non-neuroendocrine vs. neuroendocrine GI tumors: An important mechanistic distinction: In neuroendocrine tumors (carcinoids, pNETs), ectopic AVP secretion is intrinsic to the secretory phenotype of the tumor cells; production may be cyclic or fluctuating, and may co-occur with other hormonal syndromes. In non-neuroendocrine GI carcinomas, SIADH results from aberrant transcriptional activation in dedifferentiated tumor cells, is typically associated with advanced disease, and lacks the cyclic character of NET-associated SIADH [33,34,38].

An additional, frequently underappreciated mechanism in GI oncology is chemotherapy-induced SIADH: cisplatin-based regimens (commonly used in gastric and esophageal cancer) can potentiate AVP secretion or cause renal salt-wasting nephropathy. A retrospective analysis of 137 esophageal cancer patients receiving cisplatin-based chemotherapy found hyponatremia in 59%, with frank SIADH confirmed in 2% of cases [41]. This iatrogenic SIADH is biochemically identical to paraneoplastic SIADH and requires explicit exclusion of treatment-related causes before attributing hyponatremia to a paraneoplastic mechanism [42,43].

#### 3.3.3. Clinical Manifestations

Ectopic production of vasopressin results in concentrated urine despite reduced serum osmolality. Clinical manifestations include nausea, lethargy, disorientation, convulsions, or coma, especially when serum sodium declines below 120 mmol/L. SIADH may develop a slowly progressive course and is often exacerbated by treatment [5,34]. Neuropsychiatric manifestations, particularly confusion and gait instability, may precede the definitive oncological diagnosis by weeks to months, creating a critical early detection window. Based on primary cohort data, even mild chronic hyponatremia (Na^+^ 130–134 mmol/L) is associated with measurable neurological deficits, falls, and impaired attention, with clinical consequences that are disproportionate to the absolute sodium level [35,36].

#### 3.3.4. Diagnostic Approach

The diagnosis is established on laboratory abnormalities, including hyponatremia with reduced plasma osmolality (<275 mOsm/kg), elevated urine sodium (>40 mmol/L), and inappropriately concentrated urine (>100 mOsm/kg), the classical Schwartz Bartter criteria in the absence of hypovolemia, adrenal insufficiency, or hypothyroidism [5,38,42]. A critical and frequently omitted step is exclusion of glucocorticoid deficiency, which produces an identical biochemical pattern and is increasingly relevant in the era of immune checkpoint inhibitor use. Additionally, cisplatin-induced renal salt wasting must be distinguished from SIADH, as their management diverges fundamentally: SIADH requires fluid restriction, whereas salt wasting requires sodium supplementation [42,43].

#### 3.3.5. Comparative Overview of SIADH by GI Tumor Type

SIADH represents an uncommon but clinically important paraneoplastic endocrine manifestation of gastrointestinal malignancies, frequently associated with severe hyponatremia, neurological complications, and advanced disease stages. Although SIADH is classically associated with pulmonary neuroendocrine tumors, increasing evidence demonstrates that several gastrointestinal malignancies, including esophageal squamous cell carcinoma, gastric adenocarcinoma, pancreatic adenocarcinoma, and gastrointestinal neuroendocrine tumors, may exhibit aberrant arginine vasopressin (AVP) secretion or cytokine-mediated dysregulation of water homeostasis. Importantly, the underlying pathogenic mechanisms, temporal patterns of SIADH onset, associated biochemical abnormalities, therapeutic responsiveness, and prognostic implications vary considerably according to tumor type and degree of neuroendocrine differentiation. To provide a comparative overview of the principal clinical, endocrine, and therapeutic characteristics associated with SIADH across gastrointestinal malignancies, the major features reported for different gastrointestinal tumor types are summarized in Table 2.

#### 3.3.6. Therapeutic Approach and Evidence Level

Reducing fluid intake is the first-line intervention. Administration of hypertonic saline is reserved for extreme instances (Na^+^ < 120 mmol/L with neurological symptoms), with correction rates strictly limited to ≤8–10 mEq/L per 24 h to prevent osmotic demyelination syndrome [42,43]. Vasopressin receptor antagonists (vaptans), particularly tolvaptan, represent the most targeted pharmacological option: the multicenter study by Berardi et al. demonstrated that tolvaptan-treated SIADH patients had significantly shorter hospitalization and a trend toward improved overall survival compared to standard treatment [37]. Demeclocycline serves as an alternative for chronic or refractory cases [43,44]. Management of the underlying malignancy often results in resolution of SIADH [9,44]. The critical principle from primary data [35,36,37] is that sodium normalization, not merely symptomatic improvement, is the endpoint that correlates with survival benefit, and that inadequate correction is independently associated with higher mortality.

#### 3.3.7. Critical Analysis

Well supported by primary evidence: Hyponatremia is an independent negative prognostic factor in cancer patients, with median survival 2 months vs. 13.2 months (HYPNOSIS study, HR = 2.65) [36]. Failure to normalize sodium during hospitalization reduces OS (Berardi multicenter, n = 90) [37]. Tolvaptan effectively corrects sodium and shortens hospitalization [37]. Even mild hyponatremia carries measurable clinical consequences [35].

Areas of uncertainty: No primary study has specifically characterized paraneoplastic SIADH in GI malignancies with a dedicated cohort; all GI-specific data derive from case reports or retrospective series. The mechanistic distinction between truly ectopic AVP production and cytokine-mediated hypothalamic AVP release in gastric cancer has not been prospectively validated. The threshold sodium level at which correction meaningfully improves cancer outcomes remains uncertain. The HYPNOSIS study assessed it dimensionally but not as a binary threshold [36].

Key gaps: The absolute incidence of paraneoplastic (vs. chemotherapy-induced) SIADH in esophageal and gastric cancers is unknown. No prospective GI-specific SIADH registry exists. Whether tolvaptan’s survival benefit extends to GI malignancy-associated SIADH or is specific to lung cancer cannot be determined from the Berardi cohort (73% lung cancer) [37]. The contribution of chemotherapy-associated SIADH to the overall hyponatremia burden in GI oncology patients is systematically underestimated due to insufficient diagnostic workup, consistent with the 59% hyponatremia rate and only 2% confirmed SIADH in the esophageal cancer chemotherapy cohort [41].

#### 3.3.8. Case Report

Shimizu et al. (2017) presented a 66-year-old man with gastric adenocarcinoma who experienced disorientation and seizures due to severe hyponatremia (Na^+^ 112 mmol/L). The diagnostic evaluation revealed SIADH with increased urine osmolality and elevated vasopressin levels. After tolvaptan administration and initiation of chemotherapy, sodium levels normalized, and neurological symptoms improved [40].

This case illustrates four clinically relevant principles supported by primary evidence. First, the severity of hyponatremia (Na^+^ = 112 mmol/L) at presentation with frank neurological compromise reflects a common pattern in GI malignancy-associated SIADH: the gradual, insidious onset allows progressive sodium depletion before symptoms become overt, and patients are frequently encountered by clinicians only when neurological deterioration makes the hyponatremia unmissable. Earlier biochemical monitoring in patients with gastric cancer would enable detection at lower sodium levels, potentially preventing neurological sequelae. Second, the simultaneous initiation of tolvaptan and chemotherapy is mechanistically rational: tolvaptan corrects the immediate biochemical crisis while chemotherapy targets the underlying hormonal source. This two-vector approach is supported by the multicenter data from Berardi et al., which demonstrate that tumor-directed treatment combined with pharmacological sodium correction yields better outcomes than either strategy alone [37]. Third, the neurological recovery following sodium normalization demonstrates a principle confirmed by the HYPNOSIS study: the correction of hyponatremia is an active therapeutic intervention with measurable prognostic impact, not merely a surrogate marker, and should be treated with the same urgency as the oncological process itself [36]. Fourth, the rate of sodium correction must be carefully managed: the clinical imperative to rapidly correct severe symptomatic hyponatremia (Na^+^ = 112 mmol/L) must be balanced against the risk of osmotic demyelination syndrome if correction exceeds 8–10 mEq/L per 24 h. In tolvaptan-naive patients with very low baseline sodium, initiating tolvaptan at the lowest available dose (7.5 mg) with daily sodium monitoring is mandatory [42,43].

### 3.4. Non-Islet Cell Tumor Hypoglycemia

#### 3.4.1. Epidemiology and Tumor Type Distribution in GI Malignancies

Non-islet cell tumor hypoglycemia (NICTH) is a rare paraneoplastic syndrome in which tumors of non-pancreatic origin cause symptomatic hypoglycemia through overproduction of insulin-like growth factor 2 (IGF-2) or its incompletely processed precursor, “big” IGF-2 [45,46,47]. The most comprehensive quantitative data available come from a systematic analysis by Ata F et al., which synthesized 172 studies encompassing 233 patients with IGF2-mediated hypoglycemia across all tumor types [4]. Key findings directly relevant to GI oncology: fibrous tissue tumors (fibroma, fibrosarcoma) were the most common overall (n = 123, 52.8%); gastrointestinal tumors were the second most common category; and hepatocellular carcinoma represented the most frequently implicated epithelial GI malignancy. The mean age at presentation was 60.6 ± 17.1 years, with a nearly equal sex distribution. Crucially, the IGF-2:IGF-1 ratio exceeded 10 in 87.6% of patients, validating this ratio as the most operationally useful diagnostic biomarker [4].

A retrospective analysis by Fukuda et al. (2017) reviewing the glucose regulatory hormone profiles of NICTH patients, including GI tumor subtypes, further characterized the hormonal suppression pattern: insulin, C peptide, GH, and IGF-1 were uniformly low, while IGF-2 was elevated in a subset, and “big” IGF-2 was measurable by specialized assay in the majority [48]. The suppression of GH alongside IGF-1 is paradoxical, given the hypoglycemic state, which reflects autonomously elevated IGF-2 activity that suppresses the GH/IGF-1 axis via negative feedback and constitutes the biochemical signature distinguishing NICTH from insulinoma and other hypoglycemic disorders [48].

#### 3.4.2. Pathophysiological Mechanisms, Molecular Detail, and GI Specific Differences

Under normal physiological conditions, IGF-2 is produced primarily by the liver and circulates bound to IGF binding proteins (IGFBPs), predominantly IGFBP-3 and the acid-labile subunit (ALS), forming a ternary complex that prevents receptor activation. In NICTH, tumor cells produce excess pro-IGF2 (“big” IGF2), an incompletely processed precursor with a molecular weight of 10–20 kDa compared to the mature 7.5 kDa IGF2 [45,46]. “Big” IGF 2 has reduced binding affinity for IGFBP 3 and ALS, resulting in higher concentrations of biologically active free IGF 2, which activates both the IGF 1 receptor and the insulin receptor isoform A (IR A). IR A is overexpressed in fetal tissues and in malignant cells, and its activation by “big” IGF 2 produces insulin-like metabolic effects: increased glucose uptake in peripheral tissues, suppression of hepatic gluconeogenesis, and enhanced glycogen synthesis [4,45,47].

The mechanistic heterogeneity across GI tumor types is clinically significant:

Hepatocellular carcinoma (HCC): The most common GI epithelial tumor causing NICTH. HCC operates through a uniquely complex mechanism: (a) ectopic “big” IGF-2 production by tumor cells; (b) impaired hepatic gluconeogenesis due to extensive parenchymal replacement, reducing the liver’s counter-regulatory capacity; and (c) potential direct tumor glucose consumption in large, metabolically active tumors. This tripartite mechanism explains why HCC-associated NICTH is particularly refractory to medical management and has the most severe hypoglycemic episodes [45,46,49]. The case reported by Kudo et al. (2019) illustrates the dual benefit of simultaneous glucose homeostasis management and tumor volume reduction in HCC with NICTH, managed with steroid therapy and transcatheter arterial embolization (TAE) [49].

Gastrointestinal Stromal Tumors (GIST): The most important mesenchymal GI source of NICTH. GIST-associated NICTH is mediated almost exclusively through “big” IGF-2 secretion. A critical mechanistic observation from the Peker et al. case series and from multiple published GIST NICTH case reports is that imatinib mesylate (a KIT/PDGFRA inhibitor used as primary GIST therapy) can resolve hypoglycemia by reducing tumor IGF-2 secretion, demonstrating that the oncological target (KIT signaling) and the hormonal mechanism (IGF-2 overexpression) are mechanistically linked through shared downstream oncogenic pathways [50]. This observation constitutes the most compelling evidence that treating the underlying tumor is simultaneously the most effective antihypercalcemic intervention.

Colorectal carcinoma: A rare and recently documented GI source. Individual case reports have confirmed IGF-2-mediated NICTH in metastatic colorectal adenocarcinoma, typically in the context of an extensive hepatic metastatic burden that additionally compromises hepatic gluconeogenesis [4].

Esophageal squamous cell carcinoma: “Big” IGF-2 production has been reported in esophageal SCC as part of a multi-hormonal paraneoplastic picture, and may coexist with PTHrP-mediated hypercalcemia or other endocrine syndromes, creating a diagnostically complex presentation [4].

#### 3.4.3. Clinical Manifestations

Clinical presentation of NICTH in GI malignancies is characterized by recurrent fasting hypoglycemia with predominantly neuroglycopenic rather than adrenergic symptoms, confusion, disorientation, altered consciousness, seizures due to the gradual and protracted nature of glucose depletion [45,47]. The absence of the typical adrenergic alarm response (sweating, palpitations, tremor) is attributable to hypoglycemia unawareness that develops over time and to preserved catecholamine suppression in chronic hypoglycemic states. Two additional paraneoplastic features may accompany NICTH: (a) acromegaloid features (coarsening of facial features, enlarged hands/feet) due to chronic IGF-2 stimulation of GH receptors in peripheral tissues, occurring in approximately 15–20% of cases; and (b) hypophosphatemia, resulting from IGF-2-mediated phosphaturia [4,45].

#### 3.4.4. Diagnostic Approach

The minimum diagnostic panel during a documented hypoglycemic episode includes: plasma glucose, insulin, C peptide, proinsulin, beta-hydroxybutyrate, sulfonylurea screen, IGF-1, IGF-2, and IGFBP-3 [45,48]. The IGF-2:IGF-1 ratio > 10 is the most operationally reliable diagnostic criterion, present in 87.6% of confirmed cases [4], and is particularly useful when total IGF-2 levels appear within normal range (as occurs with “big” IGF-2, which may not be detected by standard immunoassays). A low IGFBP 3 alongside low IGF 1, in the context of hypoinsulinemic hypoglycemia, constitutes the biochemical fingerprint of NICTH [45,48].

#### 3.4.5. NICTH in GI Tumors: Mechanistic and Clinical Comparison

NICTH represents a rare but potentially life-threatening paraneoplastic endocrine syndrome associated with several gastrointestinal malignancies. The syndrome is most commonly mediated by tumor-derived incompletely processed insulin-like growth factor 2 (“big” IGF-2), leading to enhanced peripheral glucose utilization, suppression of counterregulatory hormones, and impaired hepatic glucose production. Although hepatocellular carcinoma and gastrointestinal stromal tumors represent the most frequently reported gastrointestinal neoplasms associated with NICTH, increasing evidence suggests that colorectal adenocarcinoma, esophageal squamous cell carcinoma, and other aggressive gastrointestinal tumors may also exhibit aberrant IGF-2-mediated endocrine activity. Importantly, the underlying molecular mechanisms, endocrine profiles, tumor characteristics, therapeutic responsiveness, and clinical outcomes vary considerably according to tumor histology and disease extent. To provide a comparative overview of the principal mechanistic, biochemical, and therapeutic characteristics associated with NICTH in gastrointestinal malignancies, Table 3 summarizes the major features reported across different gastrointestinal tumor types.

#### 3.4.6. Therapeutic Approach and Evidence Level

Glucose management is the first-line intervention in acute NICTH: continuous intravenous dextrose infusions titrated to maintain glucose levels > 70 mg/dL, supplemented by frequent oral complex-carbohydrate intake [45,47].

Glucocorticoids (prednisone 20–60 mg/day or dexamethasone 4–8 mg/day) suppress IGF-2 and “big” IGF-2 production and have the most consistent evidence base across GI tumor types. Response is typically observed within 24–48 h [48,49].

Recombinant human growth hormone (rhGH) restores IGFBP-3 and ALS levels, thereby increasing “big” IGF-2 binding and reducing free IGF-2 activity, and is most effective in combination with glucocorticoids for refractory cases.

Surgical resection, when feasible, is the only curative intervention and produces immediate resolution of hypoglycemia [4,45,50].

Imatinib is specifically indicated for KIT-expressing GIST-associated NICTH and may resolve hypoglycemia even before a substantial reduction in tumor volume [50]. Pasireotide has been reported in isolated cases as an alternative for refractory NICTH, though its evidence base remains limited to case reports [4].

Importantly, diazoxide and standard octreotide are consistently ineffective in NICTH, as they target insulin secretion rather than IGF-2 production [4].

#### 3.4.7. Critical Analysis

Well-supported by primary evidence: IGF-2:IGF-1 ratio > 10 is diagnostic in 87.6% of cases [4]. GI tumors (HCC, GIST) are the first- and second-most common epithelial and mesenchymal tumors of the GI tract [4]. Glucocorticoids reliably suppress “big” IGF-2 and restore euglycemia as a bridge therapy [33,48,49]. Imatinib resolves NICTH in GIST by targeting the upstream oncogenic pathway [50]. Tumor resection is the only curative intervention [4].

Areas of uncertainty: Standard IGF-2 immunoassays frequently underestimate “big” IGF-2, the most bioactive form, because they cross-react incompletely with the pro-form; this contributes to underdiagnosis when IGF-2 appears “normal.” The IGF-2:IGF-1 ratio is falsely low in states of severe malnutrition, cachexia, and renal failure (all common in advanced GI cancer), where both IGF-1 and IGF-2 are suppressed. No randomized controlled trial has evaluated any treatment for NICTH; all evidence derives from case reports, case series, and retrospective analyses [4].

Key gaps: The true incidence of NICTH in HCC and GIST is systematically underestimated, likely due to incomplete biochemical evaluation of hypoglycemic episodes in oncological patients. No prospective study has evaluated the prognostic impact of NICTH on outcomes in GI cancer. The role of novel anti-IGF-2 therapies (monoclonal antibodies, IGF-1R inhibitors) in unresectable NICTH remains unclear, as clinical trial data specifically in GI malignancies are lacking.

#### 3.4.8. Case Report Extended Analysis

Kudo et al. (2019) reported a patient with hepatocellular carcinoma presenting with non-islet cell tumor hypoglycemia, successfully managed with steroid therapy and transcatheter arterial embolization (TAE), demonstrating that multi-modal management combining metabolic stabilization with tumor volume reduction can achieve sustained euglycemia even in patients who are not surgical candidates [49].

Peker et al. (2012) reported a GIST patient with NICTH who achieved complete resolution of hypoglycemia following initiation of imatinib, providing direct clinical evidence that tumor-targeted therapy can eliminate the paraneoplastic metabolic syndrome by addressing its molecular root [50].

Analytical Interpretation: These two cases, taken together, illustrate a fundamental principle with broad clinical implications: the mechanistic link between the oncological target and the paraneoplastic endocrine syndrome must determine the choice of metabolic intervention. In HCC-associated NICTH, where no specific oncological target exists for the IGF-2-producing mechanism, the approach is multimodal, including steroids to suppress IGF-2 production, TAE to reduce tumor bulk, and continuous glucose support as a bridge. In GIST-associated NICTH, the presence of an actionable molecular target (KIT/PDGFRA) that is mechanistically upstream of IGF-2 overexpression means that oncological and metabolic therapies converge into a single intervention: imatinib. This mechanistic convergence is unique among GI paraneoplastic syndromes and demonstrates that the degree of tumor-target specificity directly determines the therapeutic hierarchy. Clinicians managing GIST patients with hypoglycemia should initiate imatinib as a priority, even before glucocorticoids, since addressing the root mechanism produces more durable normoglycemia than symptomatic management alone.

### 3.5. Acromegaly (Ectopic GHRH Secretion)

Acromegaly is, in the vast majority of cases, a disease of the pituitary gland. The recognition that a small subset of patients present with an identical clinical picture due to ectopic secretion of growth hormone-releasing hormone (GHRH) from extrapituitary tumors, most commonly neuroendocrine tumors of pancreatic and bronchial origin, represents one of the more fascinating examples of how GI tumors can hijack the hypothalamic–pituitary axis from a distance. Ectopic GHRH-induced acromegaly accounts for fewer than 1% of all cases of acromegaly, placing it among the rarest paraneoplastic endocrine syndromes. What makes it clinically treacherous is precisely its rarity: the phenotype is indistinguishable from pituitary acromegaly, and without a deliberate search for an extrapituitary source, patients risk undergoing unnecessary neurosurgical intervention on a normal or mildly hyperplastic pituitary gland [51,52,53].

#### 3.5.1. Epidemiology and the Pancreatic NET Connection

The most comprehensive primary dataset on this condition comes from Garby et al. (2012), who identified 21 patients with biochemically confirmed ectopic GHRH acromegaly through the French national GHRH assay registry over a 25-year period to date, the largest prospective adjacent institutional series in the literature [54]. Median plasma GHRH was markedly elevated at 548 ng/L (range 270–9779 ng/L), against a normal ceiling of 10–60 ng/L. Among GI-related sources, pancreatic NETs accounted for approximately 30–35% of all ectopic GHRH cases across the published literature, second only to bronchial carcinoids [54]. A subsequent comprehensive analysis by Zendran et al. (2022), reviewing 127 histologically confirmed cases from the global literature, found that 93% of GHRH-secreting tumors were neuroendocrine in nature, with pancreatic NETs accounting for approximately 35% and bronchial carcinoids approximately 50% of extracranial sources [55]. Colonic carcinoma as an isolated cause of ectopic GHRH has been described in the case reported by Yoshida et al. (2022), establishing that even non-neuroendocrine GI tumors can, in exceptional circumstances, acquire GHRH-secreting capacity through mechanisms that likely involve epigenetic derepression of the GHRH gene [53].

A notable and clinically important finding from the Zendran series concerns the MEN1 connection: among patients with pancreatic GHRH-secreting NETs, 76% harbored a germline MEN1 mutation [55]. This association is far from coincidental. The MEN1 gene product, menin, is a tumor suppressor that regulates chromatin remodeling and transcription factor activity; its loss facilitates aberrant expression of multiple hormonal peptide genes in pancreatic endocrine cells, including GHRH, and explains why a young patient presenting with primary hyperparathyroidism and acromegaly, the classical MEN1 phenotype, should always prompt measurement of plasma GHRH as part of the etiological investigation [54,55].

#### 3.5.2. Pathophysiology

GHRH produced ectopically by the tumor enters the systemic circulation and stimulates pituitary somatotroph cells through the GHRH receptor (GHRH R), activating adenylyl cyclase and cAMP-dependent pathways that upregulate GH gene transcription. Unlike pulsatile hypothalamic GHRH release, ectopic tumor secretion is typically non-pulsatile and continuous, producing sustained somatotroph stimulation that first leads to diffuse pituitary hyperplasia and, in some cases, to somatotroph adenoma formation [51,56]. This is a mechanistically important distinction from pituitary origin acromegaly: the pituitary gland is a secondary, reactive target rather than the primary lesion, and surgical removal of pituitary tissue will not cure the disease. The IGF-1 elevation that results from chronic GH excess mediates the somatic manifestations of acromegaly, acral enlargement, prognathism, soft tissue hypertrophy, metabolic derangements including insulin resistance and diabetes mellitus, cardiovascular remodeling, and obstructive sleep apnea [51,53,57].

#### 3.5.3. Clinical Presentation

The insidious onset of acromegaly, which typically precedes diagnosis by 7–10 years in both pituitary and ectopic forms, means that by the time a GI NET patient is recognized as having an acromegalic phenotype, significant end-organ consequences are already present. The clinical presentation, coarsening of facial features, enlargement of hands and feet, hyperhydrosis, arthralgia, and headaches, is identical in ectopic and pituitary origin disease, which is precisely why the diagnosis is so frequently delayed or misdirected. MRI of the pituitary in ectopic GHRH acromegaly may be deceptively normal, show diffuse hyperplasia mimicking a microadenoma, or, rarely, reveal a frank adenoma resulting from chronic GHRH stimulation. Any of these findings can lead to unnecessary transsphenoidal surgery and subsequent disappointment when postoperative GH and IGF 1 remain elevated [52,54]. The French nationwide series provides a striking illustration of this diagnostic trap: in a subset of patients, a pituitary lesion was identified on MRI and operated on before the ectopic source was eventually recognized [54].

#### 3.5.4. Diagnosis

The diagnostic algorithm should include, as first-line tests, measurement of IGF-1, fasting GH (with glucose suppression testing), and plasma GHRH. An elevated plasma GHRH (>300 ng/L) is the most specific marker of ectopic disease and should trigger whole body imaging, CT of the chest, abdomen, and pelvis, and ^68^Ga DOTATATE PET/CT for somatostatin receptor-expressing tumors [53,56,57]. Standard octreotide scintigraphy has been shown to have limited sensitivity in some ectopic GHRH series, and should no longer be considered adequate as a single localization tool [53,54]. In patients with features suggesting MEN1 at a young age, hyperparathyroidism, and family history, genetic testing should be integrated from the outset rather than deferred [55].

#### 3.5.5. Ectopic GHRH Acromegaly in GI Tumors: Summary of Primary Data

Ectopic growth hormone-releasing hormone (GHRH)-mediated acromegaly represents a rare endocrine paraneoplastic syndrome associated predominantly with neuroendocrine neoplasms and characterized by chronic stimulation of pituitary somatotroph hyperplasia and excessive growth hormone secretion. Among gastrointestinal tumors, pancreatic neuroendocrine tumors constitute the most frequent source of ectopic GHRH production, although foregut carcinoid tumors and exceptionally rare colorectal malignancies have also been reported. Importantly, the clinical presentation, plasma GHRH levels, imaging characteristics, somatostatin receptor expression, therapeutic responsiveness, and surgical curability vary considerably according to tumor type and extent of disease. To provide a comparative overview of the principal clinical, biochemical, radiologic, and therapeutic characteristics associated with ectopic GHRH-mediated acromegaly in gastrointestinal tumors, Table 4 summarizes the major features reported across different gastrointestinal tumor types.

#### 3.5.6. Treatment

When the tumor is resectable, surgical excision is curative. GH and IGF-1 normalize within days, and pituitary hyperplasia regresses over months [52,54]. For unresectable or metastatic GHRH-secreting tumors, long-acting somatostatin analogs (octreotide LAR, lanreotide autogel) suppress both GHRH and GH secretion and can achieve biochemical remission in somatostatin receptor-positive tumors, though responses vary with receptor expression [56,58]. Pegvisomant, a GH receptor antagonist, has been used successfully in cases where SSA fail to normalize IGF-1 [58,59]. Radiotherapy targeting residual pituitary hyperplasia is occasionally required when persistent GH excess causes ongoing morbidity after tumor resection. Overall, the prognosis of ectopic GHRH acromegaly is more favorable than that of other GI paraneoplastic syndromes, primarily because the underlying NETs, particularly well-differentiated pNETs, tend to have indolent biology and respond to multiple lines of therapy.

#### 3.5.7. Critical Appraisal

The evidence base for this syndrome is necessarily limited by its extreme rarity. The Garby et al. French series [54] remains the most important single dataset and supports several conclusions: plasma GHRH measurement is diagnostic and cost-effective; CT has approximately 86% sensitivity for tumor localization in the chest and abdomen; and surgical resection, when feasible, produces durable remission. The biological connection between MEN1 mutations and pNET GHRH secretion is well established [55], but the epigenetic mechanisms by which non-neuroendocrine GI tumors acquire GHRH expression, as in the Yoshida colonic carcinoma case [53], remain incompletely understood. Whether ectopic GHRH acromegaly is systematically underdiagnosed in GI NET patients remains an open question, given that plasma GHRH measurement is not routinely included in the endocrine workup of pNET patients.

#### 3.5.8. Case Report

Yoshida et al. described a patient with colonic carcinoma who developed overt acromegalic features, prognathism, enlarged extremities, and diaphoresis over a period of years before the underlying tumor was identified. Biochemical evaluation revealed elevated GH, elevated IGF 1, and markedly elevated plasma GHRH, with pituitary MRI showing hyperplasia but no discrete adenoma. After colonic resection, GHRH and GH levels normalized, and acromegalic features regressed [53].

This case is instructive on several levels. The years-long latency between the onset of acromegalic changes and diagnosis characteristic of the syndrome, regardless of etiology, underscores the need for metabolic vigilance in patients with known GI malignancies who report even subtle changes in shoe size, ring fit, or facial appearance. These are not trivial symptoms but measurable biological changes driven by autonomous IGF-1 activity. Second, the absence of a pituitary adenoma on MRI in the context of frank acromegalic biochemistry should, in any experienced endocrinologist’s assessment, raise the immediate possibility of ectopic GHRH rather than a missed microadenoma. The diagnostic reflex of proceeding directly to pituitary surgery in this setting without first measuring plasma GHRH represents a preventable clinical error. Third, the complete biochemical remission following tumor resection in a case of colonic carcinoma demonstrates that even non-neuroendocrine GI tumors can be the sole driver of systemic GH excess, and their removal is sufficient to restore hormonal homeostasis, provided no pituitary adenoma has formed from years of GHRH overstimulation, which is the one scenario that complicates the post-surgical picture.

### 3.6. Gynecomastia and Gonadotropin Syndromes

Gynecomastia, the benign proliferation of male breast glandular tissue, arises whenever the estrogen-to-androgen ratio at the tissue level shifts in favor of estrogen, regardless of the underlying cause. In the context of GI malignancies, this shift can be driven by two fundamentally different mechanisms: ectopic secretion of human chorionic gonadotropin (hCG) by tumor cells, which stimulates testicular aromatase and drives secondary estrogen overproduction, or direct intratumoral aromatization of androgens to estrogens, as occurs characteristically in a subset of hepatocellular carcinomas [59,60]. These two pathways lead to the same clinical endpoint, painful bilateral gynecomastia in a male cancer patient, but have different biochemical fingerprints, different tumor associations, and different implications for treatment.

The syndrome is underappreciated in clinical practice, and the reason is straightforward: gynecomastia is so common in middle-aged and elderly men for entirely mundane reasons, such as drug effects, obesity, hypogonadism, and liver disease, that its occurrence in a cancer patient rarely triggers an endocrine investigation. Yet when it develops acutely, progresses rapidly, or appears in the absence of the usual precipitating factors, it carries real diagnostic value. It may be the first, and occasionally the only, clinical signal of an otherwise occult GI malignancy.

#### 3.6.1. The hCG Pathway Ectopic Gonadotropin Secretion

Human chorionic gonadotropin is physiologically produced by placental syncytiotrophoblasts. Its ectopic production by non-trophoblastic tumors through aberrant activation of the CGB gene cluster encoding the β-subunit is the principal mechanism of paraneoplastic gynecomastia in GI cancers [59,60]. In males, circulating hCG stimulates testicular Leydig cells via the LH/hCG receptor, driving testosterone production that is then locally aromatized to estradiol in peripheral tissues. Simultaneously, elevated estrogen suppresses pituitary LH and FSH through negative feedback, creating a paradoxical hormonal pattern: high testosterone and estradiol, low LH and FSH, and very high hCG, a configuration that, in an oncological context, is essentially pathognomonic of ectopic gonadotropin secretion [59,60,61].

Within GI malignancies, ectopic hCG production has been documented in gastric adenocarcinoma, colorectal carcinoma, and in rare but well characterized cases in primary choriocarcinoma of the colon. Gastric cancer producing hCG tends to present at an advanced, metastatic stage; the case reported by Arai et al. (2010) [62] and confirmed earlier case descriptions suggest that hCG-secreting gastric tumors often harbor poorly differentiated or giant cell histological features, consistent with a more aggressive biology. Colorectal origin is rare but particularly instructive: Te Julián et al. (2015) described gynecomastia as the presenting manifestation of primary choriocarcinoma of the colon, a tumor type so unusual in this location that it was initially misdiagnosed, underscoring that hCG elevation in a male patient without obvious testicular pathology mandates a thorough GI evaluation alongside the more intuitive gonadal workup [63].

#### 3.6.2. The Aromatase Pathway Intratumoral Estrogen Synthesis

Hepatocellular carcinoma occupies a distinct mechanistic category. Rather than secreting a gonadotropin, a subset of HCC cells overexpresses the enzyme aromatase (CYP19A1), which catalyzes the intratumoral conversion of circulating androgens, primarily androstenedione and testosterone, to estrogens. This produces the unusual situation of a tumor that acts as an ectopic steroidogenic organ, generating estrogen autonomously and independently of gonadotropin stimulation [64,65]. Fibrolamellar HCC, a histologically distinct variant predominantly affecting younger patients, is particularly prone to this mechanism; the molecular basis involves amplification of a DNAJB1-PRKACA fusion gene that alters hepatocyte transcription programs and drives anomalous CYP19A1 expression [64,66]. The biochemical consequence is elevated estradiol, suppressed LH and FSH, and, importantly, normal or low hCG, which is the key differentiating feature from gonadotropin-mediated gynecomastia and should guide the investigative approach.

The broader prevalence of paraneoplastic syndromes in HCC has been quantified by a recent Romanian multicenter retrospective study by Burciu et al. (2024), which analyzed 378 consecutive HCC patients over seven years at the Timișoara County Hospital [67]. Paraneoplastic syndromes were present in 25.7% of patients, with a statistically significant male predominance (82.5% in the PNS-positive group vs. 70.6% overall, *p* = 0.0226) and a strong correlation with larger tumor size and elevated AFP levels, suggesting that endocrine paraneoplastic activity is a marker of more advanced, biologically aggressive disease rather than an incidental finding. PNS-positive patients showed a higher proportion of Child-Pugh C stage (31.8%), and hypoglycemia specifically was the only paraneoplastic manifestation associated with significantly worse survival (*p* < 0.0001). The dataset does not specifically enumerate feminization or gynecomastia separately, reflecting the ongoing lack of systematic endocrine monitoring in HCC clinical practice being a gap that warrants correction.

#### 3.6.3. Clinical Presentation and Its Diagnostic Value

Gynecomastia in a male with a GI malignancy warrants a structured hormonal evaluation rather than dismissal as a coincidental finding. The symptom itself, particularly when painful, bilateral, and progressive, has real discriminating power. A 2003 Swedish prospective cohort of 446 men operated for gynecomastia over a decade found a significantly elevated risk of subsequent esophageal cancer among those investigated, with a standardized incidence ratio suggesting a biologically plausible association [68]. While this cohort was not designed to demonstrate causation, it adds epidemiological weight to the clinical practice of investigating unexplained gynecomastia from an oncological perspective.

Clinically, the distinction between hCG-mediated and aromatase-mediated feminization should be considered at the initial evaluation, as it directly shapes the diagnostic and therapeutic approach. High hCG with elevated estradiol and suppressed LH points to gonadotropin secretion and mandates oncological imaging, including, importantly, endoscopic evaluation of the GI tract. Normal hCG with elevated estradiol and suppressed LH points to intratumoral aromatization, most commonly in HCC, and mandates hepatic imaging. In either case, urine hCG (to exclude heterophile antibody interference), testicular ultrasound, and AFP should be part of the baseline investigation [59,60,65].

#### 3.6.4. Paraneoplastic Gynecomastia in GI Malignancies: Mechanistic and Clinical Comparison

Although paraneoplastic gynecomastia remains an uncommon manifestation of gastrointestinal malignancies, increasing evidence suggests that multiple endocrine and tumor-associated mechanisms may contribute to its development. Ectopic β-human chorionic gonadotropin (β-hCG) secretion, intratumoral aromatase overexpression, altered peripheral steroid metabolism, and dysregulated hypothalamic–pituitary–gonadal signaling represent the principal pathogenic pathways implicated in this phenomenon. Importantly, the underlying endocrine profile, clinical presentation, laboratory abnormalities, and disease progression may vary considerably according to tumor type and histopathological differentiation. To provide a comparative overview of the principal mechanistic, biochemical, and clinical characteristics associated with paraneoplastic gynecomastia in gastrointestinal malignancies, the major features reported in gastric adenocarcinoma, colorectal carcinoma with choriocarcinomatous differentiation, and hepatocellular carcinoma are summarized in Table 5.

#### 3.6.5. Treatment

The most effective treatment for paraneoplastic gynecomastia is control of the underlying malignancy. Resolution of gynecomastia parallels the fall in hCG or estradiol following chemotherapy, resection, or targeted therapy [59,63]. Aromatase inhibitors (anastrozole, letrozole) represent the most pharmacologically rational symptomatic treatment for aromatase-mediated feminization in HCC, reducing intratumoral and peripheral estrogen synthesis without affecting the tumor’s hormonal biology directly [65]. Tamoxifen has been used symptomatically for the breast tissue itself, though its effect on the underlying hormonal mechanism is limited. It merits noting that systemic estrogen excess in male GI cancer patients is not merely an aesthetic concern: estrogen-driven proliferation of breast glandular tissue can, over time, create a background for oncological transformation, and chronically suppressed gonadotropins may affect bone density and quality of life in longer-surviving patients.

#### 3.6.6. Critical Appraisal

The evidence base for paraneoplastic gynecomastia in GI cancers is almost entirely case-report driven, with the partial exception of the Burciu et al. cohort [67], which provides systematic prevalence data for HCC-associated PNS overall. No study has prospectively measured hCG or sex hormone levels in consecutive GI cancer patients, meaning the true incidence of subclinical ectopic gonadotropin production detectable biochemically before gynecomastia becomes symptomatic is genuinely unknown. The molecular biology of CGB gene derepression in gastric and colorectal cancers has been studied in cell lines, but lacks prospective clinical validation. Whether hCG measurement could serve as a monitoring biomarker for hCG-secreting GI tumors analogous to its role in germ cell tumors remains a hypothesis supported by biological plausibility but not by clinical trial data.

#### 3.6.7. Case Reports

The case reported by Te Julián et al. (2015) [63] is among the most instructive in the literature on this syndrome, precisely because it concerns a choriocarcinoma of the colon that should not exist by conventional oncological logic. A 52-year-old man presented with rapidly progressive bilateral gynecomastia as his chief complaint, with a serum hCG of several thousand mIU/mL. The initial standard workup focused on testicular and mediastinal pathology; the colonic origin was identified only after a thorough GI investigation. What this case teaches the clinician is a principle of broader applicability: in any male patient with pathological hCG elevation and no apparent trophoblastic, testicular, or pulmonary source, GI endoscopy is not an optional afterthought but a mandatory diagnostic step. Choriocarcinomatous differentiation is a rare but real phenotypic transition that poorly differentiated GI tumors can undergo, and its hallmark ectopic hCG will be present in the serum long before the tumor’s unusual histology is suspected.

The case of Simões et al. (2018) [66], describing paraneoplastic feminization in hepatocellular carcinoma with documented intratumoral aromatase activity, adds a complementary lesson. The insidious onset of feminization in HCC patients, often attributed to cirrhosis-associated hypogonadism, means that an endocrinological etiology distinct from liver failure is rarely investigated. Yet the distinction matters therapeutically: cirrhosis-related gynecomastia responds poorly to aromatase inhibitors, while aromatase-mediated paraneoplastic feminization does [65]. The practical implication is that HCC patients with gynecomastia should have a full sex hormone panel, including estradiol, testosterone, LH, FSH, and hCG, measured at diagnosis, as this would simultaneously diagnose the paraneoplastic mechanism, guide the most appropriate symptomatic treatment, and potentially serve as an additional biochemical monitoring parameter alongside AFP and imaging.

### 3.7. Other Rare Endocrine Manifestations

In addition to the well-recognized paraneoplastic endocrine conditions, certain rare hormonal disturbances have been associated with gastrointestinal malignancies. Hyperthyroidism due to ectopic synthesis of thyroid-stimulating hormone (TSH)-like compounds is a rare disorder. Patients may develop characteristic thyrotoxicosis symptoms, including weight loss, tachycardia, and heat sensitivity. Gastric carcinoma and hepatocellular carcinoma have been associated in isolated cases with this condition [5,69,70].

Song et al. described the case of a 59-year-old female patient diagnosed with gastric cancer who presented with laboratory-confirmed hyperthyroidism. Thyroid imaging showed no pathological changes. Although free T4 levels were low, serum TSH levels were elevated, and tumor evaluation confirmed ectopic TSH expression. Following tumor resection, thyroid function parameters improved [69].

- Although most cases of hyperprolactinemia are associated with pituitary adenomas, it can occasionally result from ectopic prolactin production by gastrointestinal malignancies, especially neuroendocrine tumors. Patients may present with galactorrhea, amenorrhea, and hypogonadism [5,71].
- Cases with ectopic calcitonin production have been described in esophageal small-cell carcinoma and in pancreatic neuroendocrine tumors. Elevated calcitonin levels may lead to secretory diarrhea and flushing, mimicking the manifestations of patients with carcinoid syndrome [9,71].
- Nagashima et al. reported a male patient with esophageal small-cell carcinoma who experienced severe diarrhea and increased serum calcitonin levels. Octreoscan imaging detected uptake in the esophagus region. Symptoms were improved during chemoradiotherapy [71].
- The diagnosis of these uncommon illnesses requires a high level of clinical suspicion, laboratory investigations, and demonstration of ectopic hormone production. Management primarily focuses on treating the underlying tumor, which often leads to normalization of endocrine parameters.

### 3.8. Overview of Gastrointestinal Tumors

For practical clinical use, two summary tables (Table 6 and Table 7) are presented below, highlighting the relationships among gastrointestinal neoplasms, their ectopic hormone secretion, and the associated paraneoplastic endocrine syndromes.

## 4. Discussion

The recognition of PESs in patients with gastrointestinal malignancies is crucial for early diagnosis and initiation of appropriate treatment. Since these conditions frequently arise before the underlying malignancy is clinically suspected, their recognition provides an important opportunity for early cancer identification.

The recognition of paraneoplastic endocrine syndromes in patients with gastrointestinal malignancies has direct clinical relevance, particularly for earlier diagnosis and timely therapeutic intervention. As highlighted in the preceding sections, these syndromes may precede the clinical detection of the underlying tumor and can represent an initial manifestation of disease.

In routine practice, unexplained endocrine abnormalities should prompt further evaluation, especially in the absence of a known endocrine disorder. Persistent hypercalcemia, hyponatremia, hypoglycemia, Cushing-like features, or feminizing manifestations may raise suspicion of an underlying malignancy and justify a more detailed investigation. Clinical observations suggest that endocrine manifestations may occur months before tumor diagnosis in a subset of patients, supporting their role as early clinical indicators.

The diagnostic process relies on integrating biochemical, imaging, and histopathological data. Hormonal profiling remains essential for defining the clinical phenotype, while dynamic endocrine testing can help clarify ambiguous cases. Imaging techniques, including cross-sectional and functional modalities, contribute to tumor localization, and histopathological evaluation with immunohistochemical analysis is required for diagnostic confirmation.

Management is best approached in a multidisciplinary setting, with treatment strategies directed both at controlling hormonal disturbances and addressing the underlying malignancy. In addition to their diagnostic value, endocrine parameters may also be useful in monitoring disease evolution, as changes in hormone levels can precede radiological evidence of progression or recurrence.

Clinical reports indicate that up to 40% of patients with PES develop endocrine manifestations months before the underlying malignancy is identified [2,72]. Early detection of these signs enables prompt hormonal control and earlier initiation of oncologic management, with a potential positive impact on disease outcome. Post-therapy monitoring of endocrine parameters may facilitate the early detection of tumor recurrence. For instance, a renewed increase in ACTH or PTHrP levels may suggest disease relapse even before changes become apparent on imaging studies [2].

At the same time, several limitations in the current body of evidence should be acknowledged. Much of the available data derives from case reports or small series, and comprehensive analyses focused specifically on gastrointestinal malignancies remain limited. This restricts the ability to define the true incidence, clinical spectrum, and prognostic significance of these syndromes.

Further research is needed to clarify the molecular mechanisms underlying ectopic hormone production, including the contribution of altered gene expression and epigenetic regulation. The identification of reliable biomarkers is also an important objective, with potential applications for earlier detection and disease monitoring. Emerging approaches, such as circulating tumor-derived markers and advances in functional imaging, may contribute to a more accurate characterization of these conditions [73,74,75,76].

In addition, the role of immune-mediated mechanisms in the development of endocrine disturbances requires further investigation. Well-designed clinical studies are needed to better define optimal diagnostic strategies and to evaluate available therapeutic options in a more systematic manner to determine the benefits and safety profile of pharmacologic treatments such as somatostatin analogs, IGF-2 inhibitors, or glucocorticoid receptor antagonists in PESs. Additionally, randomized controlled trials may help clarify the relative value of surgical versus medical management in hormonally active gastrointestinal cancers [76,77].

## 5. Conclusions

Paraneoplastic endocrine syndromes in gastrointestinal malignancies represent a clinically relevant, although often underrecognized, aspect of gastrointestinal malignancies. Among these, hypercalcemia and syndrome of inappropriate antidiuretic hormone secretion are among the most frequently encountered and clinically significant, whereas others, such as ectopic Cushing syndrome or tumor-related hypoglycemia, tend to be less common but are often associated with more severe clinical courses and increased morbidity.

A key clinical implication is that these endocrine disturbances may precede the diagnosis of the underlying tumor and can therefore serve as early indicators of malignancy. At the same time, their presence may reflect tumor burden, biological aggressiveness, or disease progression, making them relevant not only for diagnosis but also for prognostic assessment and disease monitoring.

Effective management requires early recognition and a coordinated multidisciplinary approach, combining symptomatic treatment of hormonal imbalances with oncologic control of the underlying disease. In this context, awareness of the associations between specific gastrointestinal tumor types and particular endocrine syndromes can support a more focused diagnostic evaluation and timely intervention.

The integration of endocrine manifestations into routine oncologic evaluation has the potential to improve early detection, guide therapeutic decision-making, and enhance patient outcomes. Future research should focus on better defining the incidence of these syndromes in gastrointestinal cancers and on identifying reliable biomarkers to support earlier diagnosis and risk stratification.

## Figures and Tables

**Table 1:** Comparative Overview of Ectopic ACTH Syndrome Across Gastrointestinal Tumor Types.

Feature	Pancreatic NET	Small Bowel NET	Colorectal Carcinoma	HCC
Frequency among EAS	15.5% of NET EAS [22]	Rare	Exceptional	Exceptional
Mechanism	Ectopic ACTH (aberrant POMC)	Ectopic ACTH; cyclic/late onset	Neuroendocrine transdifferentiation	ACTH-independent cortisol; rare ectopic ACTH
Cushingoid appearance	Partial/atypical	More typical (slow-growing)	Absent (cachexia dominates)	Atypical
Typical cortisol level	Very high (midnight cortisol elevated) [23]	Moderately elevated	Very high	Variable
Hypokalemia	Severe, characteristic	Present	Present	Present
Surgical curability	Low (11%) [22]	Moderate if localized	Very low	Very low
Somatostatin receptor expression	Variable (SSR2+)	Usually SSR2+	Absent	Absent
Imaging of choice	^68^Ga DOTATATE PET/CT	^68^Ga DOTATATE PET/CT	^18^F FDG PET/CT	CT/MRI
Medical cortisol control	Ketoconazole/metyrapone + SSA	Ketoconazole/metyrapone + SSA	Ketoconazole/metyrapone	Ketoconazole/metyrapone
5-year survival (EAS)	Short (vs. bronchial carcinoid) [21,22]	Intermediate	Very short	Very short
Evidence level	Multicenter cohort [22]	Case series	Case reports	Case reports

SSA = somatostatin analogs; SSR2 = somatostatin receptor type 2; EAS = ectopic ACTH syndrome; HCC = hepatocellular carcinoma; NET = neuroendocrine tumor.

**Table 2:** Comparative Clinical and Pathophysiological Characteristics of SIADH in Gastrointestinal Malignancies.

Feature	Esophageal SCC	Gastric Adenocarcinoma	Pancreatic Adenocarcinoma	GI Neuroendocrine Tumor
Mechanism	Ectopic AVP (epigenetic de-repression)	Ectopic AVP or IL 6 mediated	Ectopic AVP (aberrant expression)	Intrinsic neuroendocrine secretion
Disease stage at SIADH	Advanced/metastatic	Variable; may be early	Advanced	Variable; may precede diagnosis
SIADH onset pattern	Acute/subacute	Subacute	Subacute	Cyclic/intermittent possible
Co-occurrence with other PES	Rare	Rare	Rare	Common (multi-hormonal secretion)
Cisplatin-related SIADH risk	High (standard chemo)	High (standard chemo)	Low	Low
Serum Na^+^ at presentation	Often <120 mmol/L	Often <120 mmol/L [40]	Variable	Variable
Response to fluid restriction	Partial	Partial	Partial	Partial
Response to tolvaptan	Yes [37]	Yes [37]	Yes	Yes
Prognosis at SIADH diagnosis	Poor (advanced stage)	Poor to intermediate	Poor	Depends on NET grade
Evidence level for GI SIADH	Case reports/series	Case reports + cohort [30,37]	Case reports [38]	Case series

SCC = squamous cell carcinoma; AVP = arginine vasopressin; IL 6 = interleukin 6; PES = paraneoplastic endocrine syndrome; NET = neuroendocrine tumor.

**Table 3:** Comparative Overview of Non-Islet Cell Tumor Hypoglycemia Across Gastrointestinal Tumor Types.

Feature	HCC	GIST	Colorectal Adenocarcinoma	Esophageal SCC
Frequency	Most common GI epithelial NICTH	Most common GI mesenchymal NICTH	Rare (case reports)	Rare (case reports)
Primary mechanism	“Big” IGF-2 + impaired gluconeogenesis + hepatic replacement	“Big” IGF-2 (KIT pathway linked)	“Big” IGF-2 + hepatic metastases	“Big” IGF-2 ± multi hormonal
Typical tumor size	Variable	Large (>10 cm)	Variable	Variable
IGF-2:IGF-1 ratio > 10	Yes [4,48]	Yes [4]	Yes [4]	Yes [4]
IGF-1	Suppressed	Suppressed	Suppressed	Suppressed
GH	Suppressed	Suppressed	Suppressed	Suppressed
Insulin/C peptide	Low	Low	Low	Low
Acromegaloid features	Rare	Possible	Rare	Rare
Surgical curability	Low (often advanced)	Yes if localized	Low (metastatic)	Low
Response to imatinib	No	Yes [50]	No	No
Response to glucocorticoids	Yes (partial) [48,49]	Yes (partial)	Yes (partial)	Yes [45,47]
Response to rhGH	Yes (adjunct)	Yes (adjunct)	Yes (adjunct)	Yes (adjunct)
Evidence level	Case series + cohort [4,48]	Case series [4,50]	Case reports [4]	Case reports [4]

HCC = hepatocellular carcinoma; GIST = gastrointestinal stromal tumor; SCC = squamous cell carcinoma; rhGH = recombinant human growth hormone.

**Table 4:** Comparative Overview of Ectopic GHRH Acromegaly Across Gastrointestinal Tumor Types.

Feature	Pancreatic NET	Colonic Carcinoma	GI Carcinoid (Foregut)
Frequency among ectopic GHRH cases	~35% [54,55]	Exceptional [40]	~5–8% [55]
MEN1 association	76% of pNET GHRH cases [55]	Not described	Rare
Tumor size at diagnosis	Variable; often >3 cm	Large	Small to medium
Plasma GHRH at presentation	Markedly elevated (>300 ng/L)	Elevated	Elevated
Pituitary MRI	Hyperplasia or normal	Hyperplasia or normal	Hyperplasia or normal
SSR expression (DOTATATE PET)	Usually positive	Variable	Usually positive
Surgical cure	Yes, if resectable [54]	Uncertain (limited data)	Yes, if resectable
Response to SSA	Good [56]	Limited data	Good
Evidence level	Multi-institutional series [54]	Case report [53]	Case series [55]

NET = neuroendocrine tumor; SSR = somatostatin receptor; SSA = somatostatin analogs.

**Table 5:** Comparative Mechanisms and Clinical Features of Paraneoplastic Gynecomastia in Gastrointestinal Malignancies.

Feature	Gastric Adenocarcinoma	Colorectal Carcinoma/Choriocarcinoma of the Colon	Hepatocellular Carcinoma (HCC)
Primary mechanism	Ectopic β-hCG → testicular aromatase → estradiol	Ectopic β-hCG (choriocarcinoma differentiation)	Intratumoral aromatase (CYP19A1 overexpression)
Typical histology	Poorly differentiated adenocarcinoma; giant cell	Choriocarcinoma/poorly differentiated adenocarcinoma	Conventional or fibrolamellar HCC
Serum β-hCG	Markedly elevated	Markedly elevated	Normal or low
Serum estradiol	Elevated	Elevated	Elevated
LH/FSH	Suppressed	Suppressed	Suppressed
Testosterone	Elevated (before peripheral aromatization)	Elevated	Low-normal (androgens consumed intratumorally)
Disease stage	Advanced/metastatic [62]	Locally advanced to metastatic [63]	Variable; often advanced [67]
Gynecomastia onset	Rapid, painful, bilateral	May precede GI diagnosis [46]	Insidious, progressive
Diagnostic test	Serum β-hCG	Serum β-hCG + colonoscopy	Estradiol, testosterone, hCG, and imaging
Treatment	Tumor-directed (hCG declines with response)	Surgical resection if possible	Aromatase inhibitors [65]; tumor treatment
Evidence level	Case reports/series [47]	Case report [46]	Cohort [67]; case reports [64,66]

LH = luteinizing hormone; FSH = follicle-stimulating hormone; HCC = hepatocellular carcinoma.

**Table 6:** GI Tumor Type → Ectopic Hormone → Syndrome.

Tumor Type	Ectopic Hormone	Paraneoplastic Syndrome
Pancreatic NET	ACTH	Cushing Syndrome
Small Bowel NET	ACTH	Cushing Syndrome
Esophageal SCC	AVP	SIADH
Gastric Carcinoma	AVP, hCG, Estrogen, TSH	SIADH, Gynecomastia, Hyperthyroidism
Hepatocellular Carcinoma	IGF-2, Estrogen, hCG, TSH	Hypoglycemia, Gynecomastia, Hyperthyroidism
Colorectal Carcinoma	PTHrP, GHRH	Hypercalcemia, Acromegaly
Gastric GIST	IGF-2	Hypoglycemia
Esophageal Small-Cell CA	Calcitonin	Diarrhea, Calcitonin Syndrome
Gastric Neuroendocrine Tumor	Prolactin	Hyperprolactinemia

NET = Neuroendocrine Tumor; SCC = Squamous Cell Carcinoma; AVP = Arginine Vasopressin (antidiuretic hormone); PTHrP = Parathyroid Hormone-related Peptide; IGF-2 = Insulin-like Growth Factor 2; GHRH = Growth Hormone-Releasing Hormone; ACTH = Adrenocorticotropic Hormone; TSH = Thyroid-Stimulating Hormone; SIADH = Syndrome of Inappropriate Antidiuretic Hormone Secretion; GIST = Gastrointestinal Stromal Tumor.

**Table 7:** Hormone → Syndrome → GI Tumor Types.

Ectopic Hormone	Paraneoplastic Syndrome	Associated GI Tumors
ACTH	Cushing Syndrome	Pancreatic NET, Small Bowel NET
AVP	SIADH	Esophageal SCC, Gastric Carcinoma
PTHrP	Hypercalcemia	Colorectal Carcinoma, Esophageal SCC
IGF-2	Hypoglycemia	HCC, Gastric GIST
GHRH	Acromegaly	Pancreatic NET, Colonic Carcinoma
hCG	Gynecomastia	HCC, Gastric Carcinoma
Estrogen-like	Gynecomastia/Feminization	HCC, Gastric Carcinoma
TSH-like	Hyperthyroidism	Gastric Carcinoma, HCC
Prolactin	Hyperprolactinemia	Gastric NET
Calcitonin	Calcitonin Syndrome	Esophageal Small-Cell CA, Pancreatic NET

ACTH = Adrenocorticotropic Hormone; AVP = Arginine Vasopressin; PTHrP = Parathyroid Hormone-related Peptide; IGF-2 = Insulin-like Growth Factor 2; GHRH = Growth Hormone-Releasing Hormone; hCG = Human Chorionic Gonadotropin; TSH = Thyroid-Stimulating Hormone; NET = Neuroendocrine Tumor; GIST = Gastrointestinal Stromal Tumor; HCC = Hepatocellular Carcinoma; SCC = Squamous Cell Carcinoma.

## Data Availability

No new data were created in this study. The data supporting this review are derived from previously published articles, which are cited throughout the manuscript.
